# Association Between Prenatal Exposure to Antipsychotics and Attention-Deficit/Hyperactivity Disorder, Autism Spectrum Disorder, Preterm Birth, and Small for Gestational Age

**DOI:** 10.1001/jamainternmed.2021.4571

**Published:** 2021-08-16

**Authors:** Zixuan Wang, Adrienne Y. L. Chan, David Coghill, Patrick Ip, Wallis C. Y. Lau, Emily Simonoff, Ruth Brauer, Li Wei, Ian C. K. Wong, Kenneth K. C. Man

**Affiliations:** 1 Research Department of Practice and Policy, UCL School of Pharmacy, London, England; 2 Department of Pharmacology and Pharmacy, Li Ka Shing Faculty of Medicine, The University of Hong Kong, Hong Kong, China; 3 Laboratory of Data Discovery for Health, Hong Kong Science Park, Hong Kong Special Administrative Region, China; 4 Groningen Research Institute of Pharmacy, Unit of PharmacoTherapy, Epidemiology and Economics, University of Groningen, Groningen, the Netherlands; 5 Department of Paediatrics and Psychiatry, Faculty of Medicine, Dentistry and Health Sciences, University of Melbourne, Melbourne, Australia; 6 Murdoch Children’s Research Institute, Melbourne, Australia; 7 Department of Paediatrics and Adolescent Medicine, Li Ka Shing Faculty of Medicine, University of Hong Kong, Hong Kong, China; 8 Department of Child and Adolescent Psychiatry, King’s College London, Institute of Psychiatry, Psychology and Neuroscience, London, England

## Abstract

**Importance:**

The risk of birth and neurodevelopmental complications with prenatal exposure to antipsychotics is unclear.

**Objective:**

To evaluate the association between prenatal antipsychotics exposure and the risk of birth and neurodevelopmental problems.

**Design, Setting, and Participants:**

This population-based cohort study included children born between January 2001 and January 2015 with follow-up to December 2019 who were identified by the Hong Kong Clinical Data Analysis and Reporting System. Pregnancies with maternal antidepressant/lithium exposure were removed. Primary analyses compared gestationally exposed and gestationally nonexposed individuals with propensity score fine stratification. Additional analyses included gestationally exposed individuals vs those with past exposure and a sibling-matched analysis to evaluate the effect of confounding by indication.

**Exposures:**

Prenatal antipsychotic exposure.

**Main Outcomes and Measures:**

Preterm birth (<37 gestational weeks), small for gestational age (birth weight <2 standard deviations below the mean for gestational age), and first diagnosis of attention-deficit/hyperactivity disorder (ADHD) and autism spectrum disorder (ASD) in children.

**Results:**

The cohorts included 333 749 mother-child pairs for ADHD (mean [SD] maternal age at delivery, 31.46 [5.03] years) and 411 251 pairs for ASD, preterm birth, and small for gestational age analyses (mean [SD] maternal age at delivery, 31.56 [5.01] years). There were 13 196 children (3.95%) with a diagnosis of ADHD, 8715 (2.12%) with ASD, 33 891 (8.24%) preterm, and 7009 (1.70%) who were small for gestational age. The weighted hazard ratio (wHR) was 1.16 (95% CI, 0.83-1.61) for ADHD and 1.06 (95% CI, 0.70-1.60) for ASD, while the weighted odds ratio (wOR) was 1.40 (95% CI, 1.13-1.75) for preterm birth and 1.36 (95% CI, 0.86-2.14) for small for gestational age when comparing gestationally exposed with gestationally nonexposed individuals. Additional analyses showed no association when comparing gestationally exposed individuals with those with past exposure (ADHD: wHR, 0.99; 95% CI, 0.60-1.61; ASD: wHR, 1.10; 95% CI, 0.58-2.08; preterm birth: wOR, 0.93; 95% CI, 0.70-1.24; small for gestational age: wOR, 1.21; 95% CI, 0.66-2.20) and in a sibling-matched analysis (ADHD: wHR, 0.41; 95% CI, 0.04-4.93; ASD: wHR, 0.90; 95% CI, 0.40-2.01; preterm birth: wOR, 1.25; 95% CI, 0.85-1.82; small for gestational age: wOR, 0.86, 95% CI, 0.32-2.31).

**Conclusions and Relevance:**

In this cohort study, the findings did not suggest that prenatal antipsychotics exposure increased the risk of ADHD, ASD, or small for gestational age. In the primary analysis, there was a small increased risk of preterm birth, but additional analyses comparing gestationally exposed individuals with those with past exposure and comparing gestationally exposed with gestationally nonexposed siblings did not support an increased risk. Given the benefits of treating psychosis during pregnancy, our findings do not support a recommendation for women to discontinue receipt of their regular antipsychotic treatment during pregnancy.

## Key Points

**Question:**

Does prenatal exposure to antipsychotics increase the risk of preterm birth, small for gestational age, attention-deficit/hyperactivity disorder (ADHD), or autism spectrum disorder (ASD)?

**Findings:**

In this cohort study of 411 251 mother-child pairs, there was not an increased risk of ADHD, ASD, preterm birth, and small for gestational age with prenatal use of antipsychotics. Maternal psychiatric disorders were associated with a significantly increased risk of ADHD and ASD, but not with preterm birth or small for gestational age in neonates.

**Meaning:**

The findings of this study suggest that there is no association between prenatal exposure to antipsychotics and ADHD, ASD, preterm birth, and small for gestational age; however, underlying maternal psychiatric disorders may be associated with the risk of ADHD and ASD in children.

## Teaser

This cohort study examines the association between prenatal antipsychotics exposure and the risk of birth and neurodevelopmental problems.

## Introduction

Antipsychotics, including first-generation and second-generation antipsychotics, are increasingly prescribed for pregnant women^1,2^; however, the safety of antipsychotic use during pregnancy remains unclear. Recent studies found that antidepressant use during pregnancy may be associated with an increased risk of neurodevelopmental disorders and birth complications, including autism spectrum disorders (ASDs),^3,4^ attention-deficit/hyperactivity disorder (ADHD),^5^ preterm birth,^6^ and small for gestational age.^6^ However, these associations may be a consequence of confounding by indication of antidepressants and/or genetic factors.^7,8,9^

There are limited studies about the association between the use of antipsychotics in pregnancy and the risk of neurodevelopmental disorders in children. A UK study with 2 to 3 years of follow-up found no association between antipsychotic use in pregnancy and the risk of neurodevelopmental disorders in infants.^10^ However, this follow-up time was not long enough to capture outcomes like ADHD, for which a diagnosis is often deferred until a child is age 5 or 6 years.^11^ Thus, any association between the risk of neurodevelopmental disorders and antipsychotic use in pregnancy remains uncertain. Moreover, a systematic review that included studies published up to 2015 reported an increased risk of preterm birth and small for gestational age^12^; however, most of the included studies had limited adjustment for confounders. Furthermore, a later study found contradictory results; therefore, it generated further uncertainties as to these associations.^13^ Randomized clinical trials were not able to evaluate the safety of antipsychotic use during pregnancy because pregnant women are typically excluded from these studies. Thus, an observational study is a practical approach to investigate these associations.^14^ This study explored the associations between prenatal antipsychotic exposure and birth and neurodevelopmental complications (preterm birth, small for gestational age, ASD, and ADHD) in children. Besides using a propensity score (PS) approach to address measured confounding from pregnancy and maternal characteristics, we also applied a sibling-matched analysis to account for unmeasured genetic and environmental factors that could be shared among the siblings; negative control analyses further supported the study conclusions.

## Methods

### Data Source and Study Design

We conducted a retrospective cohort study using the Clinical Data Analysis and Reporting System (CDARS), which contains the electronic health records of all residents (more than 7.4 million) from public hospitals and ambulatory clinics in Hong Kong.^15^ Data from CDARS have been used for various pharmacoepidemiological studies,^16,17,18^ including studies on maternal antipsychotics use^1^ and the risk of neurodevelopmental disorders because of prenatal medication use.^7^ Study protocols were approved by the institutional review board of the University of Hong Kong/Hospital Authority Hong Kong West Cluster for CDARS database research. Informed consent was waived as this study did not have patient contact and used anonymized data.

### Study Population

The study cohort included all pregnant episodes of females aged 15 to 50 years old who delivered a live birth between January 1, 2001, and December 31, 2015. Preterm birth and small for gestational age were identified and recorded at the delivery date. For ASD, all children had at least 3 years of follow-up by the end of the study period (December 31, 2018). For ADHD, we limited inclusion to deliveries between January 1, 2001, and December 31, 2013, to have at least 6 years of follow-up by the end of the study period (December 31, 2019). We defined a valid mother-child linkage as an exact match of mother and child patient identification numbers and delivery date, which are linked permanently and immediately after delivery. As a result, this linkage is highly accurate.^7^ Children without valid mother-child linkage and incomplete birth information (such as sex, birth date, gestational week, and Apgar Score) were excluded.

### Pregnancy Period

The gestational age at birth was calculated and recorded by relevant health care professionals based on ultrasonography that was performed at the first obstetric visit and directly accessed from CDARS.^7^ To identify the start and 3 trimesters of pregnancy, the last menstrual period (LMP) was estimated by date of delivery minus gestational age at birth. The pregnancy period was defined as the period between the LMP and delivery date (eFigure 1 in the Supplement). We defined any time before the LMP as the prepregnancy period. To examine the potential associations with the timing of antipsychotic use, we further separated the pregnancy period into trimesters: first trimester (0-90 days after the LMP), second trimester (91-180 days after the LMP) and third trimester (181 days after the LMP to delivery).^7,8^

### Exposure and Comparator Cohorts

Prescriptions of any antipsychotic listed in chapter 4.2.1 of the British National Formulary were extracted from the prescribing and dispensing records (eTable 1 in the Supplement). Children were considered to have been exposed prenatally if their mother received any antipsychotics during the pregnancy period (gestationally exposed). Although unlikely to be causal, previous studies showed some association between prenatal antidepressants or lithium treatment exposure and the risk of our study outcomes.^5,7,8^ Therefore, we removed the pregnancy episodes with maternal exposure to antidepressants or lithium during pregnancy to minimize the association.

Based on maternal antipsychotic use at different risk periods, we further classified the children into 3 groups: (1) those whose mothers did not use antipsychotics during pregnancy (gestationally nonexposed), within whom we further specified a subgroup as gestationally nonexposed with psychiatric disorders (coded using the *International Classification of Diseases, Ninth Revision, Clinical Modification* [*ICD-9-CM*] codes 290-319); (2) those with mothers who used antipsychotics before pregnancy but whose mothers discontinued receipt of treatment when pregnant (past exposure); and (3) those who never used antipsychotics either before or during pregnancy (never exposed), within which subgroup analyses further classified into (1) never exposed with psychiatric disorders and (2) never exposed without psychiatric disorders (eFigure 2 in the Supplement).

### Outcomes

Study outcomes were ADHD (*ICD-9-CM* code 314 or prescription for ADHD medication, namely methylphenidate or atomoxetine [British National Formulary chapter 4.4], which were the only available medications for ADHD in Hong Kong),^19^ ASD (*ICD-9-CM*: 299), preterm birth (<37 gestational weeks) and small for gestational age (birth weight <2 standard deviations below the mean for gestational age^20^). Attention-deficit/hyperactivity disorder and ASD are typically diagnosed clinically in Hong Kong in accordance with the *Diagnostic and Statistical Manual of Mental Disorders* (Fifth Edition) (*DSM-5*).^21^ Participants were followed up until the outcome onset, which was the end of the study period or death.

### Covariates

Covariates were identified for confounding adjustment. These variables included maternal age at delivery, calendar year at delivery, birth hospital, infant’s sex, parity, maternal underlying medical conditions (eg, hypertension, psychiatric disorders [*ICD-9-CM*: 290-319], epilepsy, gestational diabetes and preexisting diabetes), and socioeconomic status.

### Statistical Analyses

Hazard ratios (HRs) with a 95% CI were estimated to study the associations for neurodevelopmental outcomes using Cox proportional hazard regression models. Odds ratios (ORs) with a 95% CI were used to estimate the associations for birth outcomes by logistic regression models. Propensity score (PS) fine stratification weighting was used to address the differences in baseline covariates. The PS, the probability of receiving treatment that is conditional on the observed characteristics at baseline, can be applied to account for confounding effects in pharmacoepidemiology studies.^22,23^ We used PS fine stratification weighting for greater precision, less residual, and equivalent bias control at low exposure prevalence compared with traditional PS methods.^22,24^ In PS fine stratification weighting, the PS was used to estimate the average treatment effect by creating fine strata rather than directly calculating weights. Following stratification, weights for exposure and reference patients in all strata were subsequently calculated based on the total number of patients within each stratum, while strata with no exposure or reference patients were dropped out before weight calculation.^24^ Based on the PS distribution of the whole cohort, 150 equally sized strata were created. A robust standard error was applied to adjust for data clustering. All variables listed in the covariates section were included in the PS model.

We conducted several additional analyses to evaluate the effect of confounding by indication. We compared those with a past exposure with those never exposed***.*** An increased risk of outcomes in the children of mothers among those with a past exposure indicates confounding by indication, as the infant was not exposed to antipsychotics. Similarly, gestationally exposed individuals were compared with those with past exposure to assess whether there was a difference in the risk of study outcomes. To assess the role of maternal psychiatric disorder, we restricted comparison cohorts to those never exposed. If maternal psychiatric disorder is associated with risk of outcomes in children, this introduces the possibility of confounding by indication. To focus on the population of women who were most clearly at risk for treatment with antipsychotics during pregnancy, we also compared gestationally exposed individuals with gestationally nonexposed individuals with psychiatric disorders. A sibling-matched analysis was conducted to control for shared genetic and social confounding factors at the family level. Stratified Cox/logistic regression with a separate stratum for each family identified by the mother’s unique identification number was used. Only inconsonant sibling pairs for maternal antipsychotic use and study outcomes could contribute to the estimates.

Subgroup analyses and sensitivity analyses were conducted to test the validity of the initial analyses (eAppendix 1 in the Supplement). A *P* value of <.05 was considered statistically significant in all statistical analyses. SAS, version 9.4 (SAS Institute) and Stata, version 15 (StataCorp), were used for data management and analysis.

## Results

**Figure 1.**
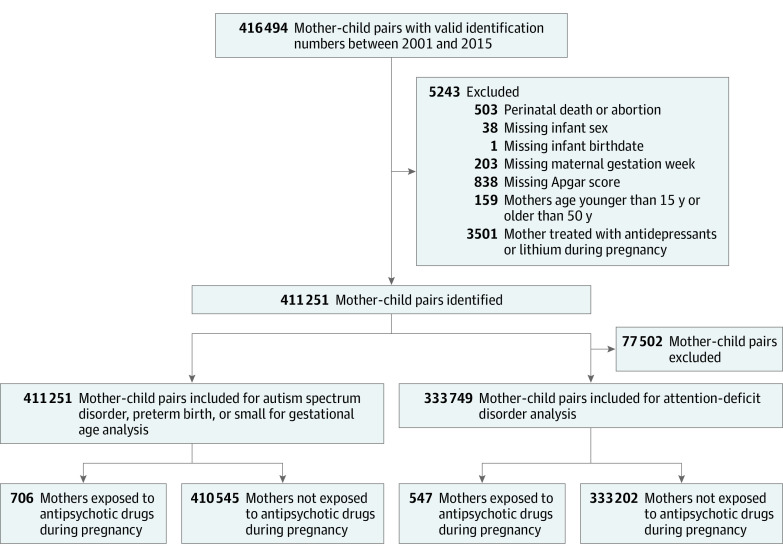
Flowchart of Mother-Child Pair Identification

**Figure 2.**
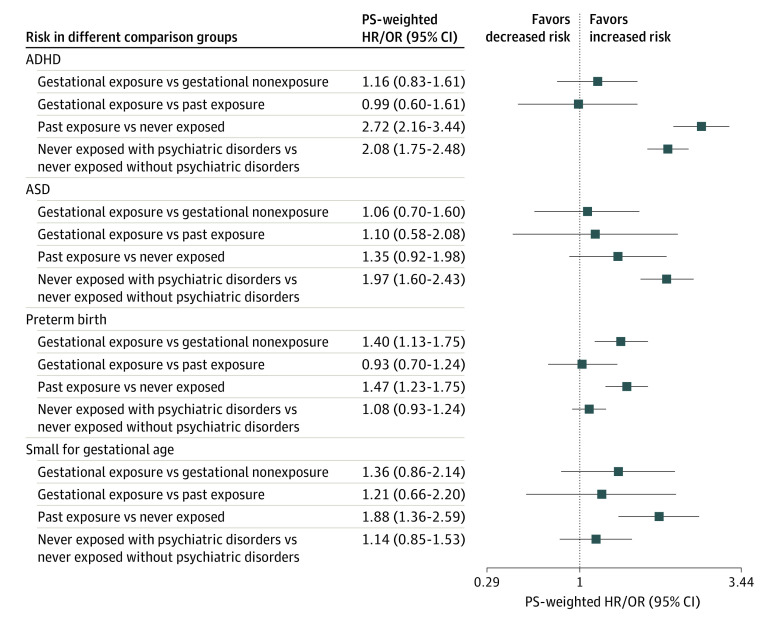
Propensity Score (PS)–Weighted Results From Analyses of Different Comparisons ADHD indicates attention-deficit/hyperactivity disorder; ASD, autism spectrum disorder; HR, hazard ratio; OR, odds ratio.

Our final cohorts included 411 251 pairs of mother-child records for ASD, preterm birth, and small for gestational age analyses and 333 749 pairs for ADHD analyses (Figure 1). The Table summarizes the patient characteristics. Overall, 706 children (0.17%) were prenatally exposed to antipsychotics between 2001 and 2015, and 27 (3.82%), 92 (13.03%), and 19 (2.69%) received a diagnosis of ASD, preterm birth, and small for gestational age, respectively. While 547 (0.16%) were exposed to antipsychotics during pregnancy between 2001 and 2013, 45 (8.23%) developed ADHD. Covariate balances were achieved after PS weighting, with all standardized differences less than 10% (eTable 2 in the Supplement). When comparing gestationally exposed with gestationally nonexposed individuals, the PS-weighted HRs (wHRs) or ORs (wORs) were 1.16 (95% CI, 0.83-1.61) for ADHD, 1.06 (95% CI, 0.70-1.60) for ASD, 1.40 (95% CI, 1.13-1.75) for preterm birth, and 1.36 (95% CI, 0.86-2.14) for small for gestational age (Figure 2; eTable 3 in the Supplement).

**Table.** Characteristics of Children and Their Mothers

Characteristics	No./total No. (%)							
	ADHD		ASD		Preterm birth		Small for gestational age	
	Exposed group	Nonexposed group	Exposed group	Nonexposed group	Exposed group	Nonexposed group	Exposed group	Nonexposed group
Any antipsychotic	45/547 (8.23)	13 151/333 202 (3.95)	27/706 (3.82)	86 88/410 545 (2.12)	92/706 (13.03)	33 799/410 545 (8.23)	19/706 (2.69)	6990/410 545 (1.70)
Different drug classes								
Only FGAs	33/345 (9.57)	NA	16/405 (3.95)	NA	59/405 (14.57)	NA	11/405 (2.72)	NA
Only SGAs	7/122 (5.74)		3/199 (1.51)		28/199 (14.07)		3/199 (1.51)	
**Children**								
Follow-up time, mean (SD), y	10.45 (3.55)	10.37 (3.26)	8.23 (4.09)	8.30 (3.78)	NA	NA	NA	NA
Boy	32/289 (11.07)	102 24/172 942 (5.91)	24/381 (6.30)	7415/212 990 (3.48)	52/381 (13.65)	18 745/212 990 (8.80)	7/381 (1.84)	2578/212 990 (1.21)
Girl	13/258 (5.04)	2927/160 260 (1.83)	3/325 (0.92)	1273/197 555 (0.64)	40/325 (12.31)	15054/197 555 (7.62)	12/325 (3.69)	4412/197 555 (2.23)
Pregnancy								
Singleton	540/547 (98.72)	32 2730/333 202 (96.86)	691/706 (97.88)	39 6997/410 545 (96.70)	691/706 (97.88)	396 997/410 545 (96.70)	691/706 (97.88)	39 6997/410 545 (96.70)
Multiple	7/547 (1.28)	10 472/333 202 (3.14)	15/706 (2.12)	13 548/410 545 (3.30)	15/706 (2.12)	13 548/410 545 (3.30)	15/706 (2.12)	13 548/410 545 (3.30)
Timing of Apgar score <7, min								
1	31/547 (5.67)	11 072/333 202 (3.32)	42/706 (5.95)	13 424/410 545 (3.27)	42/706 (5.95)	13 424/410 545 (3.27)	42/706 (5.95)	13 424/410 545 (3.27)
5	3/547 (0.55)	1063/333 202 (0.32)	4/706 (0.57)	1395/410 545 (0.34)	4/706 (0.57)	1395/410 545 (0.34)	4/706 (0.57)	1395/410 545 (0.34)
Birth weight, g								
<1500	12/547 (2.19)	3729/333 202 (1.12)	13/706 (1.84)	4576/410545 (1.11)	13/706 (1.84)	4576/410 545 (1.11)	13/706 (1.84)	4576/410 545 (1.11)
1500-2499	49/547 (8.96)	25 446/333 202 (7.64)	72/706 (10.20)	31 450/410 545 (7.66)	72/706 (10.20)	31 450/410 545 (7.66)	72/706 (10.20)	31 450/410 545 (7.66)
≥2500	486/547 (88.85)	304 027/333 202 (91.24)	621/706 (87.96)	374 519/410 545 (91.22)	621/706 (87.96)	374 519/410 545 (91.22)	621/706 (87.96)	374 519/4105 45 (91.22)
Gestation wk								
<33	17/547 (3.11)	5899/333 202 (1.77)	19/706 (2.69)	7142/410 545 (1.74)	19/706 (2.69)	7142/410 545 (1.74)	19/706 (2.69)	7142/410 545 (1.74)
33-36	48/547 (8.78)	21 852/333 202 (6.56)	73/706 (10.34)	26 657/410 545 (6.49)	73/706 (10.34)	26 657/410 545 (6.49)	73/706 (10.34)	26 657/410 545 (6.49)
>36	482/547 (88.12)	305 451/333 202 (91.67)	614/706 (86.97)	376 746/410 545 (91.77)	614/706 (86.97)	376 746/410 545 (91.77)	614/706 (86.97)	376 746/410 545 (91.77)
**Mothers**								
Maternal age at delivery, mean (SD), y	31.81 (5.70)	31.46 (5.03)	31.93 (5.85)	31.56 (5.01)	31.93 (5.85)	31.56 (5.01)	31.93 (5.85)	31.56 (5.01)
Parity								
0	289/547 (52.83)	174 625/333 202 (52.41)	391/706 (55.38)	215 922/410 545 (52.59)	391/706 (55.38)	215 922/410 545 (52.59)	391/706 (55.38)	215 922/410 545 (52.59)
1	146/547 (26.69)	124 389/333 202 (37.33)	178/706 (25.21)	153 470/4105 45 (37.38)	178/706 (25.21)	153 470/410 545 (37.38)	178/706 (25.21)	153 470/410 545 (37.38)
2	69/547 (12.61)	26 872/333 202 (8.06)	86/706 (12.18)	32 496/410 545 (7.92)	86/706 (12.18)	32 496/410 545 (7.92)	86/706 (12.18)	32 496/410 545 (7.92)
≥3	43/547 (7.86)	7316/333 202 (2.20)	51/706 (7.22)	8657/410 545 (2.11)	51/706 (7.22)	8657/410 545 (2.11)	51/706 (7.22)	8657/410 545 (2.11)
Maternal underlying conditions								
Preexisting diabetes	3/547 (0.55)	814/333202 (0.24)	9/706 (1.27)	1071/410 545 (0.26)	9/706 (1.27)	1071/410 545 (0.26)	9/706 (1.27)	1071/410 545 (0.26)
Gestational diabetes	101/547 (18.46)	36 688/333202 (11.01)	135/706 (19.12)	47 142/410 545 (11.48)	135/706 (19.12)	47 142/410 545 (11.48)	135/706 (19.12)	47 142/410 545 (11.48)
Hypertension	20/547 (3.66)	12 135/333 202 (3.64)	34/706 (4.82)	15 259/41 0545 (3.72)	34/706 (4.82)	15 259/410 545 (3.72)	34/706 (4.82)	15 259/410 545 (3.72)
Psychiatric disorder	396/547 (72.39)	2664/333 202 (0.80)	509/706 (72.10)	3714/410 545 (0.90)	509/706 (72.10)	3714/410 545 (0.90)	509/706 (72.10)	3714/410 545 (0.90)
Epilepsy	4/547 (0.73)	511/333 202 (0.15)	9/706 (1.27)	655/41 0545 (0.16)	9/706 (1.27)	655/410 545 (0.16)	9/706 (1.27)	655/410 545 (0.16)
Median household income, HK$								
<19 300	133/547 (24.31)	68 510/333202 (20.56)	225/706 (31.87)	115 323/410 545 (28.09)	225/706 (31.87)	115 323/410 545 (28.09)	225/706 (31.87)	115 323/410 545 (28.09)
19 300-21 999	169/547 (30.90)	944 61/333 202 (28.35)	200/706 (28.33)	105 272/410 545 (25.64)	200/706 (28.33)	105 272/410 545 (25.64)	200/706 (28.33)	105 272/410 545 (25.64)
22 000-25 999	143/547 (26.14)	85 435/333 202 (25.64)	168/706 (23.80)	96 003/41 0545 (23.38)	168/706 (23.80)	96 003/410 545 (23.38)	168/706 (23.80)	96 003/41 0545 (23.38)
≥26 000	102/547 (18.65)	84 796/333 202 (25.45)	113/706 (16.01)	93 947/41 0545 (22.88)	113/706 (16.01)	93 947/410 545 (22.88)	113/706 (16.01)	93947/410 545 (22.88)

Abbreviations: ADHD, attention-deficit/hyperactivity disorder; ASD, autism spectrum disorder; FGA, first-generation antipsychotics; HK$, Hong Kong dollar; NA: not applicable; SGAs: second-generation antipsychotics.

### Other Comparisons

**Figure 3.**
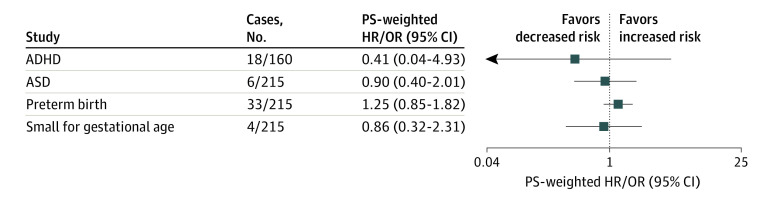
Propensity Score (PS)–Weighted Results From Sibling-Matched Analysis ADHD indicates attention-deficit/hyperactivity disorder; ASD, autism spectrum disorder; HR, hazard ratio; OR, odds ratio.

Additional analyses were conducted to evaluate the effect of confounding by indication. All comparison group results are found in Figure 2 and Figure 3 as well as eTable 4 in the Supplement.

#### Gestationally Exposed vs Past Exposure

There was no statistically significant difference between gestational vs past maternal exposure in the risk of ADHD (wHR, 0.99; 95% CI, 0.60-1.61), ASD (wHR, 1.10; 95% CI, 0.58-2.08), preterm birth (wOR, 0.93; 95% CI, 0.70-1.24), or small for gestational age (wOR, .21; 95% CI, 0.66-2.20). Results suggested no difference in the risk of all outcomes when comparing gestationally exposed vs past exposure.

#### Past Exposure vs Never Exposed

The risk of ADHD (wHR, 2.72; 95% CI, 2.16-3.44), preterm birth (wOR, 1.47; 95% CI, 1.23-1.75), or small for gestational age (wOR, 1.88; 95% CI, 1.36-2.59) was statistically significantly increased in the maternal past exposure group vs those never exposed. There was no evidence to support an increased risk of ASD (wHR, 1.35; 95% CI, 0.92-1.98).

#### Never Exposed With Psychiatric Disorders vs Never Exposed Without Psychiatric Disorders

When the analysis was restricted to mothers who had never used antipsychotics, the risk of neurodevelopmental disorders was higher in mothers with psychiatric disorders (ADHD: wHR, 2.08; 95% CI, 1.75-2.48; ASD: wHR, 1.97; 95% CI, 1.60-2.43). There is no evidence to support the risk of preterm birth (wOR, 1.08; 95% CI, 0.93-1.24) or small for gestational age (wOR, 1.14; 95% CI, 0.85-1.53).

### Sibling-Matched Analysis

The sibling-matched analysis contained 23 308 mothers with 48 275 children in the ADHD cohort and 40 756 mothers with 85 257 infants in the ASD, preterm birth, and small for gestational age cohort. Eighteen of 160 exposed (11.25%) and 1906 of 48 115 unexposed siblings (3.96%) received a diagnosis of ADHD; 6 of 215 exposed (2.79%) and 1630 of 85 042 unexposed siblings (1.92%) had ASD; 33 of 215 exposed (14.88%) and 9270 of 85 042 unexposed children (10.90%) were preterm; and 4 of 215 exposed (1.86%) and1598 of 85 042 unexposed children (1.88%) were small for gestational age. There was no significantly increased risk of ADHD (wHR, 0.41; 95% CI, 0.04-4.93), ASD (wHR, 0.90; 95% CI, 0.40-2.01), preterm birth (wOR, 1.25; 95% CI, 0.85-1.82), or small for gestational age (wOR, 0.86; 95% CI, 0.32-2.31) in siblings of mothers who were gestationally exposed to antipsychotics compared with gestationally nonexposed (Figure 3; eTable 5 in the Supplement).

**Figure 4.**
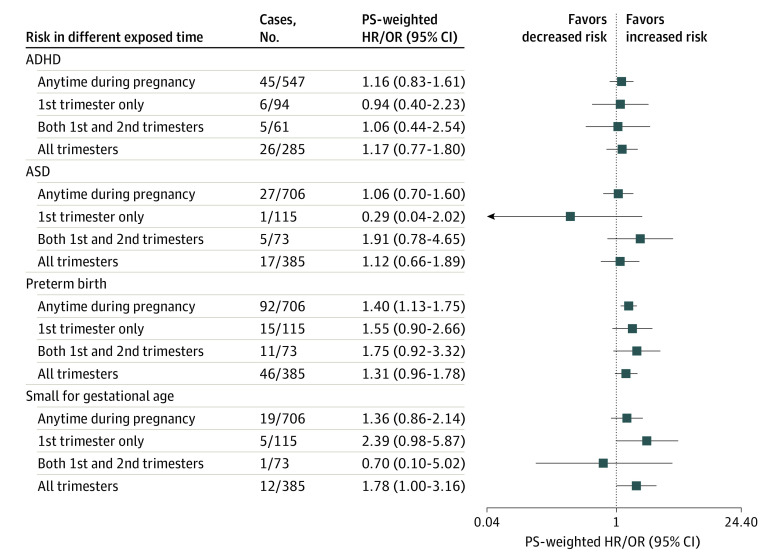
Propensity Score (PS)–Weighted Results in Comparing Those Gestationally Exposed to Antipsychotics With Gestationally Nonexposed Individuals Different outcomes of interest in comparing those gestationally exposed to antipsychotics with gestationally nonexposed individuals in different exposed times. ADHD indicates attention-deficit/hyperactivity disorder; ASD, autism spectrum disorder; HR, hazard ratio; OR, odds ratio.

Most exposed women had long-term antipsychotic treatment throughout the entire pregnancy period; the evidence did not support any association between gestational antipsychotic exposure at different trimesters and the risk of study outcomes (Figure 4; eTable 3 in the Supplement). Interpretations of the subgroup analyses and sensitivity analyses are shown in eAppendix 2 and eTables 6 and 7 in the Supplement.

## Discussion

From the primary analysis, we can only rule out more than a 1.61-, 1.60-, 1.75-, and 2.14-fold higher risk for ADHD, ASD, preterm birth, and small for gestational age, respectively, between children with and without prenatal exposure to antipsychotics. However, considering the analyses of the negative control (Figure 2; eTable 4 in the Supplement), sibling-matched (Figure 3; eTable 5 in the Supplement), and several sensitivity and subgroup analyses (eTables 6 and 7 in the Supplement), our study results do not suggest an increased risk of ADHD, ASD, preterm birth, and small for gestational age that is associated with prenatal exposure to antipsychotics.

However, among pregnant women who were never exposed to antipsychotics, children born to mothers with psychiatric disorders had a higher risk of neurodevelopmental disorders (ASD and ADHD), but not negative birth outcomes (preterm birth and small for gestational age) compared with those with mothers without psychiatric disorders. Furthermore, the risk was the same for unexposed siblings and exposed siblings. These results suggest that maternal psychiatric disorders are associated with a higher risk of neurodevelopmental disorders rather than gestational exposure to antipsychotic drugs. Moreover, gestational exposure to antipsychotic drugs is unlikely to pose a significant risk of preterm birth and small for gestational age in children. Although our estimates had relatively wide intervals, given the benefits of antipsychotic treatment, our findings do not support a recommendation for women to discontinue receipt of their regular antipsychotic treatment during pregnancy.

Previous studies were limited by including relatively low numbers of women who were prescribed antipsychotics during pregnancy, inadequate duration of follow-up, inadequate mother-child record linkage, and poorly specified exposure time in the study design.^14^ Previous studies have usually addressed confounding by health and lifestyle factors and concomitant medication using simple matching methods.^10,13^ This study not only addressed these limitations, but has also expanded on them by using several advanced approaches (sibling-matched analyses and a series of sensitivity analyses with a negative control) to address potential confounding factors and strengthen the reliability of our conclusion.

Our findings are consistent with those of Petersen et al^10^, who focused on general developmental disorders in children and had a limited follow-up time.^10^ Petersen et al^10^ and our study found no evidence to support an association between antipsychotic medication use during pregnancy and an increased risk of neurodevelopmental disorders in children. Our results are also consistent with another Canadian population-based study^13^ that found no evidence to support an increased risk of preterm birth or small for gestational age in children with antipsychotic exposure during pregnancy. Petersen et al^10^ and Vigod et al^13^ did not report any difference between the antipsychotic classes or sex of the child.^10,13^ However, our study shows that antipsychotic use during pregnancy was not associated with an additional risk of ADHD, ASD, preterm, or small for gestational age compared with those with a past exposure or those never exposed (with and without psychiatric disorders). It is important in future studies to identify whether a pregnant woman with active psychotic disorders would benefit from taking antipsychotics, including effective control of psychotic symptoms and minimal adverse effects to herself, offspring, and her family.

### Clinical Implications and Recommendations

Our study results suggest that if pregnant women have a clinical need for antipsychotics (including first-generation and second-generation antipsychotics), clinicians should not stop administering regular treatment because of a fear of birth outcomes with ADHD, ASD, preterm birth, and small for gestational age. There has been a lengthy debate about a possible association between in-utero exposure to psychotropic medications and neurodevelopmental disorders or birth complications in children. Patients and clinicians have encountered difficulties in treating women with severe affective and psychotic disorders when trying to conceive and during pregnancy. Previous studies found that parents with psychiatric disorders are more likely to have offspring with neurodevelopmental disorders, such as ASD^25^; clinicians should observe on a case-by-case basis. Major adverse effects can occur if ongoing treatment is discontinued abruptly or antipsychotics are withheld during pregnancy. This study’s findings provide data to guide clinicians in decision-making.

### Strengths and Limitations

Our study has several strengths. To our knowledge, this is the first study to use a population-based database to identify the association between prenatal antipsychotics exposure and the risk of ADHD or ASD in children separately with adequate follow-up time. We also evaluated the association using stratification by drug class and timing of exposure. To identify the exposures, we used electronic dispensing and prescribing records, which are free from recall bias. Moreover, we used complementary negative control analyses and sibling-matched analyses to address the possibility of confounding by indication. Deterministic linkage records between mothers and children, as well as explicit gestation age records, were available in CDARS, which enhanced the accuracy and reliability of our findings.^14^

There are also several limitations. First, CDARS only includes public health care medical records; data from private hospitals and medical practitioners cannot be captured. However, children with neurodevelopmental disorders require comprehensive long-term treatment; thus, in Hong Kong, they usually receive care from the public sector.^26^ Second, poor antipsychotic adherence among patients with psychiatric disorders is common,^27^ and we cannot confirm whether patients took the prescribed medication, which may affect the accuracy of the results. We addressed the use misclassification using at least 2 prescriptions or a 56-day coverage time of prescriptions records and 1-week and 2-week prescription extension in the sensitivity analyses, which had similar results to our primary analyses. Third, CDARS is not primarily used for research purposes. Factors that may affect the risk of study outcomes, such as body mass index (calculated as weight in kilograms divided by height in meters squared), smoking, and alcohol consumption status are not recorded in CDARS. However, we used a complementary negative control and sibling-matched analyses, which are unlikely to affect the interpretation of our findings. Fourth, as analyses were conducted using Hong Kong population data, it is unclear whether the results are generalizable. Although our study results are consistent with previous western studies,^10,13^ future research should be conducted in other settings. Fifth, the data extraction for this study was restricted based on the data minimization principle; therefore, we only have the relevant variables as presented in this study. Information on the indications for nonspontaneous cases was not available in the data set. Thus, we were unable to conduct the analysis on other birth defect outcomes. As suggested in a recent review,^28^ further studies on birth defects or malformation should be conducted. Lastly, large samples with adequate statistical power are necessary for sibling-matched analyses.^29^ Although we included all Hong Kong sibling samples, the sample size has insufficient power to detect small differences. Also, as exposed patients were limited, we lacked adequate power for a dose-response analysis of each drug.

## Conclusions

This study’s findings do not suggest an association between prenatal exposure to antipsychotics and the risk of ADHD, ASD, preterm birth, and small for gestational age. Given that maternal psychiatric disorders may increase neurodevelopmental disorder risk in children,^25^ clinicians should inform individual patients about the benefits and potential risks of using antipsychotics during pregnancy.

## Notes

Supplement. eFigure 1. Pregnancy period definition eTable 1. List of antipsychotics in the British National Formulary Chapter 4.2.1 eFigure 2. Control groups definition eAppendix 1. Subgroup analyses and sensitivity analyses eTable 2. Standardized mean differences before and after PS weighting in different covariates eTable 3. Results from analysis comparing gestational-exposed to antipsychotics with gestational non-exposed eTable 4. Results from analyses of different comparisons eTable 5. Results from the sibling-matched analysis eAppendix 2. Interpretation of subgroup analyses and sensitivity analyses eTable 6. Results from analyses of sub-groups eTable 7. Results from analyses of sensitivity analyses
