# Supplementary material for: Association Between Prenatal Exposure to Antipsychotics and Attention-Deficit/Hyperactivity Disorder, Autism Spectrum Disorder, Preterm Birth, and Small for Gestational Age
Source: JAMA Intern Med. 2021 Aug 16;181(10):1332–40. doi: 10.1001/jamainternmed.2021.4571 (PMC8369381; doi:10.1001/jamainternmed.2021.4571)
Supplement: Supplement. — eFigure 1. Pregnancy period definition eTable 1. List of antipsychotics in the British National Formulary Chapter 4.2.1 eFigure 2. Control groups definition eAppendix 1. Subgroup analyses and sensitivity analyses eTable 2. Standardized mean differences before and after PS weighting in different covariates eTable 3. Results from analysis comparing gestational-exposed to antipsychotics with gestational non-exposed eTable 4. Results from analyses of different comparisons eTable 5. Results from the sibling-matched analysis eAppendix 2. Interpretation of subgroup analyses and sensitivity analyses eTable 6. Results from analyses of sub-groups eTable 7. Results from analyses of sensitivity analyses [file jamainternmed-e214571-s001.pdf]

Supplemental Online Content

Wang Z, Chan AYL, Coghill D, et al. Association between prenatal exposure to antipsychotics and attention-deficit/hyperactivity disorder, autism spectrum disorder, preterm birth, and small for gestational age. *JAMA Intern Med*. Published online August 16, 2021. doi:10.1001/jamainternmed.2021.4571

eFigure 1. Pregnancy period definition
eTable 1. List of antipsychotics in the British National Formulary Chapter 4.2.1
eFigure 2. Control groups definition
eAppendix 1. Subgroup analyses and sensitivity analyses
eTable 2. Standardized mean differences before and after PS weighting in different covariates
eTable 3. Results from analysis comparing gestationally exposed to antipsychotics with gestationally non-exposed
eTable 4. Results from analyses of different comparisons
eTable 5. Results from the sibling-matched analysis
eAppendix 2. Interpretation of subgroup analyses and sensitivity analyses
eTable 6. Results from analyses of sub-groups
eTable 7. Results from analyses of sensitivity analyses

This supplemental material has been provided by the authors to give readers additional information about their work.

© 2021 Wang Z et al. *JAMA Internal Medicine.*

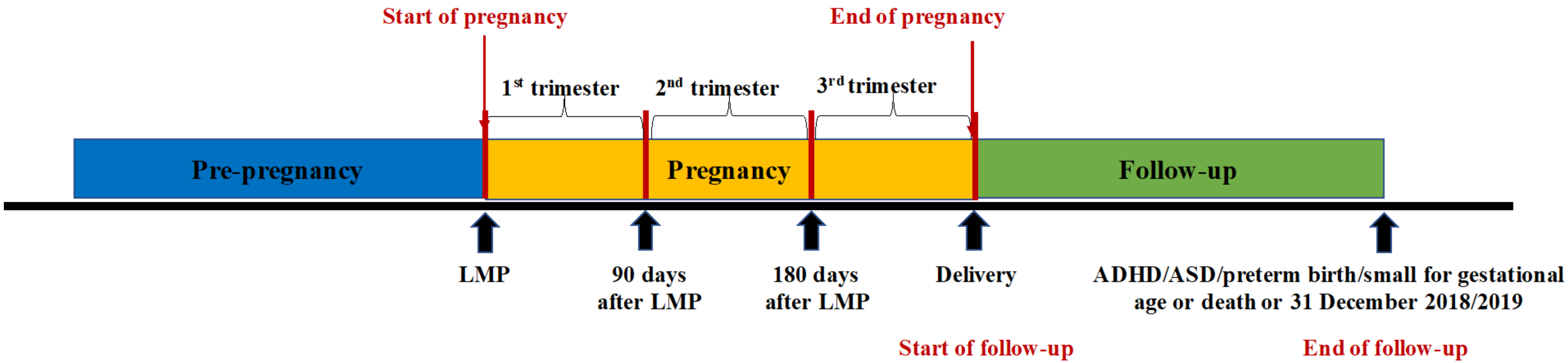

eFigure1. Pregnancy period definition

LMP: last menstrual period; ADHD: attention-deficit/hyperactivity disorder; ASD: autism spectrum disorder

© 2021 Wang Z et al. *JAMA Internal Medicine.*

eTable 1. List of antipsychotics in the British National Formulary Chapter 4.2.1

List of drugs
Amisulpride
Aripiprazole
Benperidol
Cariprazine
Chlorpromazine Hydrochloride
Chlorprothixene
Clozapine
Flupentixol Hydrochloride
Fluphenazine Hydrochloride
Haloperidol
Levomepromazine Hydrochloride
Levomepromazine Maleate
Loxapine Succinate
Lurasidone
Melperone Hydrochloride
Olanzapine
Paliperidone
Pericyazine
Perphenazine
Pimozide
Promazine Hydrochloride
Quetiapine
Risperidone
Sulpiride
Thioridazine
Trifluoperazine
Ziprasidone Hydrochloride
Zotepine
Zuclopenthixol Acetate
Zuclopenthixol Hydrochloride

© 2021 Wang Z et al. *JAMA Internal Medicine.*

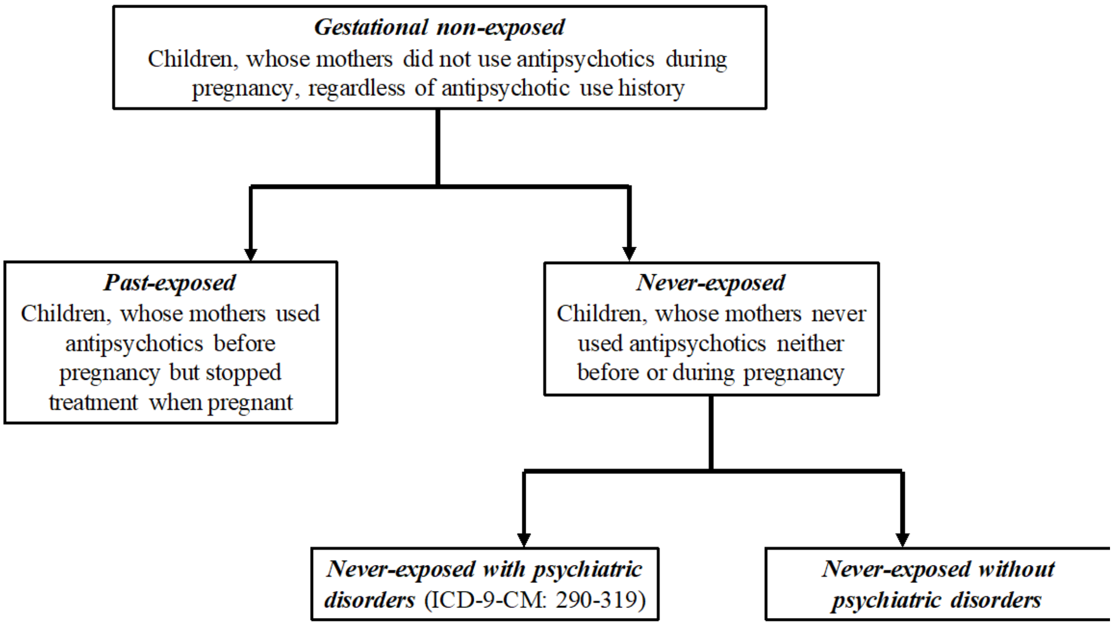

eFigure2. Control groups definition

ICD-9-CM: International Classification of Diseases, 9th Revision, Clinical Modification

© 2021 Wang Z et al. *JAMA Internal Medicine.*

eAppendix 1: Subgroup analyses and sensitivity analyses

Subgroup analyses

Three subgroup analyses were conducted:

1) To further identify the risk of study outcomes in exposed individuals with different drug classes, we classified FGAs only and SGAs only subgroups.

2) As neurodevelopmental outcomes are more common in boys than girls,[1] to ascertain gender difference, we conducted additional subgroup analyses in boy and girl groups respectively.

3) As mode of delivery is available in our dataset, we conducted a subgroup analysis by defining spontaneous preterm birth as preterm cases with normal spontaneous delivery, and others as non-spontaneous preterm birth.

Sensitivity analyses

Four sensitivity analyses were conducted to test the validity of the initial analyses:

1) To assess the impact of potential exposure misclassification, we restricted inclusion to mothers who had received at least two prescriptions during pregnancy and extended the prescription period by 7 and 14 days; we also limited women who had at least 56 days coverage time of prescriptions to test the effect of length of the prescription period.

2) To investigate the potential clustering effect of children who were born to the same mother, we restricted the analyzes to the first pregnancy episode only.

3) History of preterm birth is a strong predictor of future preterm birth; to further adjust for this factor, we added a sensitivity analysis by removing the first pregnancy episode and included history of preterm birth as an additional covariate.

4) We conducted an additional sensitivity analysis that did not exclude offspring born to women with antidepressant and lithium use during pregnancy.

References:

1. Association AP. Diagnostic and statistical manual of mental disorders (DSM-5®). American Psychiatric Pub; 2013.

© 2021 Wang Z et al. *JAMA Internal Medicine.*

eTable 2. Standardized mean differences before and after PS weighting in different covariates

Covariate categories		ADHD		ASD/preterm birth/small for gestational age	
		Before PS weighting	After PS weighting	Before PS weighting	After PS weighting
		Standardized difference (%)	Standardized difference (%)	Standardized difference (%)	Standardized difference (%)
**Calendar year at delivery**		-8.9	-5	-0.2	-0.5
**Maternal age at delivery**		6.7	-0.8	6.9	-1
**Median household income (HK$)**	<19300	9	1.2	8.3	1.2
	19300-21999	5.6	-1.2	6.1	-0.7
	22000-25999	1.1	-0.5	1	-0.4
	>=26000	-16.5	0.8	-17.4	-0.2
**Mother with hypertension**	Yes	0.1	-0.4	5.4	-0.9
**Mother with psychiatric disorders**	Yes	222.1	7.7	219.6	9.7
**Mother with pre-existing diabetes**	Yes	4.8	0.5	11.6	2.2
**Mother with epilepsy**	Yes	8.7	0.8	13.2	0.6
**Mother with gestational diabetes mellitus**	Yes	21.1	1.2	21.3	-0.2
**Baby gender**	Girl	-1.9	-0.1	-4.2	0.5
**Parity**	0	0.9	0.7	5.6	1.5
	1	-23	-0.9	-26.5	-0.4
	2	15	-0.1	14.2	-0.9
	>=3	26.1	0.3	24.4	-1.2

PS: propensity score; ADHD: attention-deficit/hyperactivity disorder; ASD: autism spectrum disorder; HK: Hong Kong; NA: not applicable

© 2021 Wang Z et al. *JAMA Internal Medicine.*

eTable 3. Results from analysis comparing gestationally exposed to antipsychotics with gestational non-exposed

Different exposed time	N in exposed	N in non-exposed	Crude estimate	PS weighted estimate
	N/Total (%)	N/Total (%)	HR/OR, 95% CI	HR/OR, 95% CI
**ADHD**				
Anytime during pregnancy	45/547	13151/333202	**2.10**	1.16
	8.23	3.95	**(1.57-2.82)**	(0.83-1.61)
1st trimester only	6/94	13151/333202	1.77	0.94
	6.38	3.95	(0.80-3.92)	(0.40-2.23)
Both 1st trimester and 2nd trimester	5/61	13151/333202	2.07	1.06
	8.20	3.95	(0.86-4.96)	(0.44-2.54)
All trimesters	26/285	13151/333202	**2.34**	1.17
	9.12	3.95	**(1.60-3.44)**	(0.77-1.80)
**ASD**				
Anytime during pregnancy	27/706	8688/410545	**1.86**	1.06
	3.82	2.12	**(1.27-2.71)**	(0.70-1.60)
1st trimester only	1/115	8688/410545	0.42	0.29
	0.87	2.12	(0.06-3.00)	(0.04-2.02)
Both 1st trimester and 2nd trimester	5/73	8688/410545	3.17	1.91
	6.85	2.12	(1.32-7.61)	(0.78-4.65)
All trimesters	17/385	8688/410545	**2.21**	1.12
	4.42	2.12	**(1.37-3.55)**	(0.66-1.89)

(continued)

© 2021 Wang Z et al. *JAMA Internal Medicine*.

eTable 3. Results from analysis comparing gestationally exposed to antipsychotics with gestational non-exposed (continued)

**Different exposed time**	**N in exposed**	**N in non-exposed**	**Crude estimate**	**PS weighted estimate**
	**N/Total (%)**	**N/Total (%)**	**HR/OR, 95% CI**	**HR/OR, 95% CI**
**Preterm birth**				
Anytime during pregnancy	92/706	33799/410545	**1.67**	**1.40**
	13.03	8.23	**(1.34-2.08)**	**(1.13-1.75)**
1st trimester only	15/115	33799/410545	1.67	1.55
	13.04	8.23	(0.97-2.88)	(0.90-2.66)
Both 1st trimester and 2nd trimester	11/73	33799/410545	**1.98**	1.75
	15.07	8.23	**(1.04-3.76)**	(0.92-3.32)
All trimesters	46/385	33799/410545	**1.51**	1.31
	11.95	8.23	**(1.11-2.06)**	(0.96-1.78)
**Small for gestational age**				
Anytime during pregnancy	19/706	6990/410545	**1.60**	1.36
	2.69	1.70	**(1.01-2.52)**	(0.86-2.14)
1st trimester only	5/115	6990/410545	**2.62**	2.39
	4.35	1.70	**(1.07-6.43)**	(0.98-5.87)
Both 1st trimester and 2nd trimester	1/73	6990/410545	0.80	0.70
	1.37	1.70	(0.11-5.77)	(0.10-5.02)
All trimesters	12/385	6990/410545	**1.86**	**1.78**
	3.12	1.70	**(1.04-3.30)**	**(1.00-3.16)**

ADHD: attention-deficit/hyperactivity disorder; ASD: autism spectrum disorder; N: number; PS: propensity score; HR: hazard ratio; OR: odds ratio; CI: confidence interval

© 2021 Wang Z et al. *JAMA Internal Medicine*.

eTable 4. Results from analyses of different comparisons

Comparison groups	ADHD		ASD		Preterm birth		Small for gestational age	
	Crude estimate	PS weighted estimate	Crude estimate	PS weighted estimate	Crude estimate	PS weighted estimate	Crude estimate	PS weighted estimate
	HR, 95% CI	HR, 95% CI	HR, 95% CI	HR, 95% CI	OR, 95% CI	OR, 95% CI	OR, 95% CI	OR, 95% CI
**Gestationally exposed vs gestational non-exposed with psychiatric disorders**	1.00 (0.70-1.42)	1.09 (0.73-1.62)	0.98 (0.63-1.54)	0.90 (0.55-1.48)	**1.46 (1.09-1.94)**	1.23 (0.92-1.65)	1.47 (0.82-2.63)	1.25 (0.70-2.23)
**Gestationally exposed vs past exposure**	0.81 (0.56-1.17)	0.99 (0.60-1.61)	1.50 (0.88-2.55)	1.10 (0.58-2.08)	1.15 (0.87-1.52)	0.93 (0.70-1.24)	0.87 (0.50-1.53)	1.21 (0.66-2.20)
**Gestationally exposed vs never-exposed with psychiatric disorders**	1.03 (0.73-1.44)	0.83 (0.55-1.25)	0.96 (0.63-1.48)	0.93 (0.58-1.50)	**1.60 (1.23-2.08)**	**1.31 (1.01-1.70)**	1.45 (0.84-2.49)	1.41 (0.81-2.48)
**Gestationally exposed vs never-exposed without psychiatric disorders**	**2.15 (1.60-2.88)**	**2.16 (1.59-2.93)**	**1.88 (1.29-2.75)**	**1.87 (1.26-2.77)**	**1.67 (1.34-2.09)**	**1.50 (1.20-1.87)**	**1.60 (1.02-2.53)**	1.55 (0.98-2.44)
**Past exposure vs never-exposed**	**2.69 (2.15-3.36)**	**2.72 (2.16-3.44)**	1.25 (0.86-1.81)	1.35 (0.92-1.98)	**1.46 (1.23-1.74)**	**1.47 (1.23-1.75)**	**1.84 (1.33-2.54)**	**1.88 (1.36-2.59)**
**Past exposure vs never-exposed with psychiatric disorders**	1.27 (0.96-1.69)	1.08 (0.76-1.54)	0.65 (0.42-0.99)	0.73 (0.46-1.15)	**1.40 (1.12-1.75)**	**1.45 (1.15-1.81)**	**1.66 (1.07-2.57)**	**1.46 (0.95-2.22)**
**Past exposure vs never-exposed without psychiatric disorders**	**2.70 (2.16-3.38)**	**2.72 (2.16-3.44)**	1.25 (0.86-1.82)	1.36 (0.93-1.99)	**1.46 (1.23-1.74)**	**1.47 (1.23-1.75)**	**1.84 (1.33-2.54)**	**1.88 (1.36-2.60)**
**Never-exposed with psychiatric disorders vs never-exposed without psychiatric disorders**	**2.11 (1.78-2.50)**	**2.08 (1.75-2.48)**	**1.94 (1.58-2.39)**	**1.97 (1.60-2.43)**	1.05 (0.91-1.21)	1.08 (0.93-1.24)	1.11 (0.83-1.48)	1.14 (0.85-1.53)

ADHD: attention-deficit/hyperactivity disorder; ASD: autism spectrum disorder; PS: propensity score; HR: hazard ratio; OR: odds ratio; CI: confidence interval; vs: versus

© 2021 Wang Z et al. *JAMA Internal Medicine*.

eTable 5. Results from the sibling-matched analysis

Outcome	Crude estimate<br>HR/OR, 95% CI	PS weighted estimate<br>HR/OR, 95% CI
**ADHD**	**3.05 (1.92-4.85)**	0.41 (0.04-4.93)
**ASD**	1.52 (0.69-3.39)	0.90 (0.40-2.01)
**Preterm birth**	1.43 (0.98-2.08)	1.25 (0.85-1.82)
**Small of gestational age**	0.99 (0.37-2.67)	0.86 (0.32-2.31)

ADHD: attention-deficit/hyperactivity disorder; ASD: autism spectrum disorder; PS: propensity score; HR: hazard ratio; OR: odds ratio; CI: confidence interval

© 2021 Wang Z et al. *JAMA Internal Medicine.*

eAppendix 2. Interpretation of the subgroup analyses and sensitivity analyses

Subgroup analyses

Subgroup analyses by FGAs or SGAs showed no significantly increased risk of ADHD/ASD/small for gestational age. However, an increased risk of preterm birth was found in both FGAs (wOR: 1.57, 95% CI: 1.19-2.07) and SGAs subgroup (wOR: 1.59, 95% CI: 1.07-2.37). No difference was found between prenatal exposure to different drug classes for all study outcomes (eTable 6).

For boys, antipsychotic use during pregnancy was significantly associated with preterm birth (wOR: 1.56, 95% CI: 1.16-2.10), but not with risk of ADHD/ASD/small for gestational age. Lack of evidence could demonstrate an increased risk of either preterm birth or ADHD/ASD/small for gestational age in girls. Compared to girls, boys had a higher risk for ADHD or ASD but a lower risk of small for gestational age, while no difference was found for preterm birth (eTable 6).

An increased risk of spontaneous preterm birth was found (wOR: 1.59, 95% CI: 1.16-2.16), with no evidence to demonstrate a higher risk of non-spontaneous preterm birth (wOR: 1.24, 95% CI: 0.92-1.66, eTable 6).

Sensitivity analyses

The results of the sensitivity analysis for at least two prescriptions, at least 56 days exposure, only first pregnancies, 7-day prescription extension and 14-day prescription extension were similar to the main analyses, i.e., non-statistical significance in ADHD/ASD/small for gestational age cohort, but statistical significance in preterm birth cohort (eTable 7).

An increased risk of preterm birth/small for gestational age was found in the sensitivity analysis that does not exclude offspring born to women with antidepressant and lithium use during pregnancy. However, no evidence supports the higher risk for ADHD or ASD (eTable 7).

After additionally adjusting history of preterm birth, there was no evidence to demonstrate an association between prenatal exposure to antipsychotics and the risk of preterm birth (eTable 7).

© 2021 Wang Z et al. *JAMA Internal Medicine.*

eTable 6. Results from analyses of sub-groups

Sub-groups	ADHD		ASD		Preterm birth		Small for gestational age	
	Crude estimate	PS weighted estimate	Crude estimate	PS weighted estimate	Crude estimate	PS weighted estimate	Crude estimate	PS weighted estimate
	HR, 95% CI	HR, 95% CI	HR, 95% CI	HR, 95% CI	OR, 95% CI	OR, 95% CI	OR, 95% CI	OR, 95% CI
**Only FGAs**	**2.31**	1.29	**1.76**	1.03	**1.90**	**1.57**	1.61	1.45
	**(1.65-3.25)**	(0.88-1.87)	**(1.08-2.88)**	(0.60-1.78)	**(1.44-2.51)**	**(1.19-2.07)**	(0.88-2.94)	(0.79-2.63)
**Only SGAs**	1.69	0.85	0.88	0.46	**1.83**	**1.59**	0.88	0.81
	(0.81-3.53)	(0.37-1.92)	(0.29-2.71)	(0.15-1.45)	**(1.22-2.72)**	**(1.07-2.37)**	(0.28-2.76)	(0.26-2.54)
**FGAs vs SGAs**	1.36	1.20	2.10	3.96	1.04	1.42	1.82	1.93
	(0.60-3.07)	(0.41-3.56)	(0.61-7.25)	(0.87-18.04)	(0.64-1.69)	(0.84-2.39)	(0.50-6.61)	(0.50-7.51)
**Boys**	**1.93**	1.16	**1.91**	1.10	**1.64**	**1.56**	1.53	1.47
	**(1.36-2.73)**	(0.79-1.69)	**(1.28-2.85)**	(0.71-1.72)	**(1.22-2.20)**	**(1.16-2.10)**	(0.72-3.23)	(0.70-3.12)
**Girls**	**2.71**	1.26	1.43	0.72	**1.70**	1.23	1.68	1.35
	**(1.57-4.68)**	(0.67-2.35)	(0.46-4.44)	(0.22-2.36)	**(1.22-2.37)**	(0.88-1.72)	(0.94-2.99)	(0.75-2.40)
**Boys vs Girls**	**2.37**	**2.62**	**7.36**	**5.80**	1.13	1.24	0.49	**0.37**
	**(1.24-4.52)**	**(1.14-6.04)**	**(2.22-24.45)**	**(1.68-20.00)**	(0.72-1.75)	(0.79-1.95)	(0.19-1.26)	**(0.15-0.93)**
**Spontaneous preterm**	NA	NA	NA	NA	**1.69**	**1.59**	NA	NA
					**(1.24-2.30)**	**(1.16-2.16)**		
**Non-spontaneous preterm**	NA	NA	NA	NA	**1.65**	1.24	NA	NA
					**(1.23-2.22)**	(0.92-1.66)		

ADHD: attention-deficit/hyperactivity disorder; ASD: autism spectrum disorder; PS: propensity score; HR: hazard ratio; OR: odds ratio; CI: confidence interval; FGAs: first-generation antipsychotics; SGAs: second-generation antipsychotics; vs: versus; NA: not applicable

© 2021 Wang Z et al. *JAMA Internal Medicine*.

eTable 7. Results from analyses of sensitivity analyses

Comparison groups	ADHD		ASD		Preterm birth		Small for gestational age	
	Crude estimate	PS weighted estimate	Crude estimate	PS weighted estimate	Crude estimate	PS weighted estimate	Crude estimate	PS weighted estimate
	HR, 95% CI	HR, 95% CI	HR, 95% CI	HR, 95% CI	OR, 95% CI	OR, 95% CI	OR, 95% CI	OR, 95% CI
**At least 2 prescriptions gestationally exposed vs gestational non-exposed**	**2.29 (1.71-3.08)**	1.24 (0.89-1.73)	**1.85 (1.24-2.76)**	1.00 (0.64-1.56)	**1.72 (1.37-2.17)**	**1.42 (1.13-1.79)**	1.53 (0.93-2.51)	1.36 (0.83-2.24)
**At least 56 days prescription gestationally exposed vs gestational non-exposed**	**2.06 (1.50-2.82)**	1.11 (0.78-1.57)	**1.86 (1.25-2.78)**	1.02 (0.65-1.58)	**1.65 (1.31-2.09)**	**1.37 (1.08-1.73)**	**1.71 (1.07-2.73)**	1.56 (0.97-2.49)
**Gestationally exposed (only first pregnancies) vs gestational non-exposed**	**1.75 (1.16-2.64)**	1.01 (0.65-1.57)	1.61 (0.97-2.68)	1.04 (0.60-1.79)	**1.63 (1.22-2.18)**	**1.37 (1.03-1.84)**	0.92 (0.46-1.85)	0.89 (0.44-1.79)
**Gestationally exposed (extend Rx period by 7 days) vs gestational non-exposed**	**2.13 (1.60-2.85)**	1.19 (0.86-1.65)	**1.84 (1.26-2.68)**	1.04 (0.68-1.57)	**1.65 (1.33-2.06)**	**1.39 (1.12-1.73)**	**1.58 (1.00-2.50)**	1.34 (0.85-2.11)
**Gestationally exposed (extend Rx period by 14 days) vs gestational non-exposed**	**2.10 (1.58-2.81)**	1.19 (0.86-1.65)	**1.82 (1.25-2.65)**	1.03 (0.68-1.56)	**1.66 (1.33-2.06)**	**1.38 (1.11-1.72)**	1.57 (0.99-2.47)	1.35 (0.85-2.13)
**Gestationally exposed vs gestational non-exposed [a]**	**2.41 (1.96-2.97)**	1.23 (0.97-1.56)	**1.99 (1.51-2.62)**	1.10 (0.81-1.49)	**1.73 (1.47-2.03)**	**1.44 (1.23-1.69)**	**1.68 (1.21-2.35)**	**1.59 (1.14-2.22)**
**Gestationally exposed vs gestational non-exposed [b]**	NA	NA	NA	NA	**1.71 (1.21-2.39)**	1.02 (0.72-1.44)	NA	NA

[a] analysis does not exclude offspring born to women with antidepressant and lithium; [b] analysis removing first pregnancy episodes and including history of preterm birth as an additional covariate

ADHD: attention-deficit/hyperactivity disorder; ASD: autism spectrum disorder; PS: propensity score; HR: hazard ratio; OR: odds ratio; CI: confidence interval; vs: versus; Rx: prescription

© 2021 Wang Z et al. *JAMA Internal Medicine*.
